# Intra- and inter-subject variability of femoral growth plate stresses in typically developing children and children with cerebral palsy

**DOI:** 10.3389/fbioe.2023.1140527

**Published:** 2023-02-24

**Authors:** Willi Koller, Basílio Gonçalves, Arnold Baca, Hans Kainz

**Affiliations:** ^1^ Department of Biomechanics, Kinesiology and Computer Science in Sport, Centre for Sport Science and University Sports, University of Vienna, Vienna, Austria; ^2^ Neuromechanics Research Group, Centre for Sport Science and University Sports, University of Vienna, Vienna, Austria; ^3^ Vienna Doctoral School of Pharmaceutical, Nutritional and Sport Sciences, University of Vienna, Vienna, Austria

**Keywords:** femoral growth plate, cerebral palsy, femoral deformities, growth plate, musculoskeletal simulation, finite element analysis, osteogenic index, personalized modelling

## Abstract

Little is known about the influence of mechanical loading on growth plate stresses and femoral growth. A multi-scale workflow based on musculoskeletal simulations and mechanobiological finite element (FE) analysis can be used to estimate growth plate loading and femoral growth trends. Personalizing the model in this workflow is time-consuming and therefore previous studies included small sample sizes (N < 4) or generic finite element models. The aim of this study was to develop a semi-automated toolbox to perform this workflow and to quantify intra-subject variability in growth plate stresses in 13 typically developing (TD) children and 12 children with cerebral palsy (CP). Additionally, we investigated the influence of the musculoskeletal model and the chosen material properties on the simulation results. Intra-subject variability in growth plate stresses was higher in cerebral palsy than in typically developing children. The highest osteogenic index (OI) was observed in the posterior region in 62% of the TD femurs while in children with CP the lateral region was the most common (50%). A representative reference osteogenic index distribution heatmap generated from data of 26 TD children’s femurs showed a ring shape with low values in the center region and high values at the border of the growth plate. Our simulation results can be used as reference values for further investigations. Furthermore, the code of the developed GP-Tool (“Growth Prediction-Tool”) is freely available on GitHub (https://github.com/WilliKoller/GP-Tool) to enable peers to conduct mechanobiological growth studies with larger sample sizes to improve our understanding of femoral growth and to support clinical decision making in the near future.

## 1 Introduction

During growth, bones of mammals do not only grow in size but also the geometry adapts according to the predominant loading conditions (Arkin and Katz, 1956; Rauch, 2005; Carter and Beaupré, 2007; Mirtz et al., 2011; Mellon and Tanner, 2012). Altered forces acting on bones can lead to various skeletal deformities. In humans, femoral deformities are common in children with cerebral palsy (CP) but also in children without neurological disorders (Bobroff et al., 1999; Morrell et al., 2002; Beals, 2008). These children are born with typical bony geometry, but in many patients the femoral neck shaft angle (NSA) and anteversion angle (AVA) does not decrease during growth as in typically developing (TD) children (Fabry et al., 1973; Bobroff et al., 1999; Robin et al., 2008). The pathological femoral geometry alters moment arms of muscles (Arnold et al., 2008) which influences muscle recruitment strategy (Kainz et al., 2021b), resulting in altered joint contact forces which are often associated with joint pain (Mackay et al., 2021). Altered movement strategies and joint loading subsequently lead to further progressive disabilities with walking impairments being one of the most profound (Rethlefsen et al., 2017). The altered loading on the growth plate is assumed to be responsible for the pathological femoral growth, yet this assumption remains to be confirmed.

Multi-scale simulations based on musculoskeletal (MSK) simulations and mechanobiological finite element (FE) analysis are used to estimate growth plate loading and femoral growth trends, i.e. the change of the femoral NSA and the AVA due to the forces acting on it (Shefelbine and Carter, 2004; Carriero et al., 2011; Yadav et al., 2016; Yadav et al., 2017; Kainz et al., 2020). First, three-dimensional gait analysis data is collected and MSK simulations are performed to estimate muscle and joint contact forces. Subsequently, these forces are used as loading conditions in a FE model of the femur to quantify the stresses at the growth plate. Based on the estimated shear and hydrostatic stresses at the growth plate the osteogenic index (OI) can be defined, which determines the subject-specific growth rate due to mechanical loading (Carter and Wong, 1988). The mechanical growth rate is added to the biological growth rate which is determined by biological factors, e.g. genetics and nutrients (Stevens et al., 1999). In regions with high OI, bone growth is more likely to occur, whereas in regions with negative OI, growth will be inhibited (Yadav et al., 2016). With these analyses, the development of pathological femoral growth could be identified at an early stage and simple and non-invasive interventions (e.g., orthoses, physical therapy) could potentially normalize bone growth and prevent the necessity of severe corrective osteotomies at a later stage.

Hip joint contact forces (HCF) and subject-specific growth plate geometry are the two main factors influencing growth plate stresses and femoral growth predictions. HCF have been shown to be the main biomarker which determines femoral bone growth (Carriero et al., 2011; Kainz et al., 2020; Yadav et al., 2021). Kainz et al. (2021a) recently showed that the orientation of the HCF in the sagittal plane can differentiate between children with CP who are likely to have typical and pathological femoral growth. HCF are influenced by the subject-specific gait pattern and femoral geometry (Kainz et al., 2020; Kainz et al., 2021b; Modenese et al., 2021). Yadav et al. (2016) showed that the growth plate shape and location affect the stress distribution within the growth plate and therefore change the OI. These studies highlight the importance of accounting for the subject-specific gait pattern and using subject-specific MSK and FE models for mechanobiological growth simulations.

All previous femoral mechanobiological growth studies were based either on a generic FE model of the femur (i.e., generic femoral morphology and growth plate shape and location) (Carriero et al., 2011; Kainz et al., 2020; 2021a) or include very small sample sizes (N < 4) (Yadav et al., 2016; Yadav et al., 2017; Yadav et al., 2021). The reason for this might be the time-consuming generation of personalized FE models. The mechanobiological model requires hexahedral elements aligned with the growth plate. The creation of such a hexahedral mesh from irregular 3D volumes of the femur is challenging and time-consuming. Furthermore, the measured material properties reported in literature (Linde et al., 1985; Rho et al., 1993) for bone vary substantially. Previous studies (Carriero et al., 2011; Yadav et al., 2016; Yadav et al., 2017; Kainz et al., 2020; Kainz et al., 2021a; Yadav et al., 2021) used material property values on the lower bounds of the reported range. Moreover, the generalization of the findings from previous studies was limited due to small sample sizes and the lack of comprehensive reference dataset for TD children. Hence, a reference data set of OI values from TD children obtained with subject-specific MSK and FE models is needed to quantify the typical OI variability within TD children and facilitate an appropriate interpretation of the findings for future femoral growth studies.

This study included the following two clinical aims: 1) quantify and compare the OI in a reference dataset of TD children and children with CP, 2) quantify the intra- and inter-subject variability of the OI. Additionally, our study included the following two technical aims: 3) evaluate the influence of the chosen MSK model, i.e. generic-scaled versus personalized femoral geometry, and 4) evaluate the effect of the chosen material properties on the simulation results. Furthermore, we analyzed and compared the shape, the location and the orientation of the growth plates between groups (CP and TD). Considering that the variability in CP gait is higher compared to TD gait (Kurz et al., 2012; Davies and Kurz, 2013), we hypothesized that the intra-subject variability in the OI is higher in children with CP compared to TD children. In addition, we hypothesized that the chosen MSK model will influence the OI distribution, whereas the material properties will mainly alter the magnitude but not the distribution of the OI. A toolbox based on freely available software, which enables the generation of the personalized FE model and perform the multi-scale simulations in a simple and comprehensive way was developed and used to address our aims. Magnetic resonance imaging (MRI) and three-dimensional gait analysis (3DGA) data of twelve children with CP and thirteen TD children were used to generate subject-specific FE models and quantify the OI based on different input data and model properties.

## 2 Materials and methods

### 2.1 Data collection

MRI and 3DGA data including marker trajectories and ground reaction forces of twelve children diagnosed with CP (10.4 ± 3.8 years old, height: 133.6 ± 16.1 cm, mass: 30.1 ± 10.8 kg) and thirteen TD children (10 ± 2.2 years old, height: 144.5 ± 8.5 cm, mass: 36.8 ± 9.5 kg) were analyzed for this study. All participants walked without walking aids and with a self-selected speed. The data of all CP children and three TD children was captured during a previous study (Kainz et al., 2017) while the data of the remaining ten TD children was additionally collected for the purpose of this study. Ethics approval was obtained from the local ethics committees (University of Vienna, reference number 00578). Data collection of the retrospective analyzed data (CP children and three TD children) is described in detail in Kainz et al. (2017).

MRI images of the additionally recorded data (ten TD children) were collected using a 3T magnetic resonance scanner (MAGNETOM Vida, Siemens, Berlin/Munich, Germany) with a T1 vibe sequence with a voxel size of 0.8 × 0.8 × 0.7 mm. 3DGA-data for these ten TD children were captured on the same day as the MRI images using a 12 camera motion capture system (Vicon Motion Systems, Oxford, UK). The used marker set during the motion capturing was based on the Plug-in-Gait marker set (Kadaba et al., 1990; Davis et al., 1991) with additional clusters of three markers on each thigh and shank segment and an additional marker at the 5th metatarsal head of each foot. Simultaneously, ground reaction forces were acquired using five force plates (Kistler Instrumente, Winterthur, Switzerland). All children performed several gait trials with a self-selected walking speed. Marker trajectories were captured, labelled, and filtered (Butterworth 4th order, 6 Hz low-pass filter) in Nexus 2.12.1 (Vicon Motion System, Oxford, United Kingdom).

### 2.2 Segmentation of MRIs and quantification of geometrical features

3D Slicer 4.13 (Fedorov et al., 2012) was used to segment MRI images. Each femur was split into five parts similar to previous studies (Kainz et al., 2020)—the proximal trabecular bone, the growth plate, the cortical bone of the shaft, the bone marrow and the distal trabecular bone. STL-files of all parts and additionally a file containing the full femur were exported. The STAPLE-Toolbox of Modenese and Renault (2021) was used to identify the femoral head and the epicondyles representing the hip and the knee joint axis using the “GIBOC-Femur” and “GIBOC-Cylinder” algorithms, respectively. If “GIBOC-Cylinder” algorithm failed to fit a cylinder through both epicondyles, “GIBOC-Ellipsoids” algorithm was used to fit ellipsoids through medial and lateral epicondyles. The hip joint center and knee joint axis were required to transform the femur into the OpenSim coordinate system.

The diaphysis of the femur was defined by removing 20% off the top and bottom of the femur. Then, the principal inertia axis of the remaining part was calculated to identify the shaft axis. The neck axis was defined by fitting a least-squares cylinder through surface nodes of the femoral neck. The longitudinal axis of this cylinder was constrained to pass through the femoral head center. The AVA was calculated as the angle between the neck axis and the medial-lateral knee axis obtained from STAPLE-Toolbox (Modenese and Renault, 2021) in the transverse plane. The NSA was computed as the angle between the neck axis and shaft axis in 3D space.

### 2.3 MSK simulations and loading for FE models

Two models, i.e., generic-scaled and personalized, were created for each participant and used to perform MSK simulations with OpenSim 4.2 to estimate muscle and joint contact forces acting on the femur (Delp et al., 2007; Steele et al., 2012). For the generic-scaled models, the generic ‘gait2392’ OpenSim model (Delp et al., 2007) with locked metatarsophalangeal joints was linearly scaled to fit to the participants’ anthropometry based on the location of surface markers (Kainz et al., 2017). For the personalized model, the Torsion Tool (Veerkamp et al., 2021) was used to modify the femoral geometry in the ‘gait2392’ model to match each child’s NSA and AVA before scaling the model. The maximum isometric muscle forces of all models were scaled based on the ratio of the body mass between the participant’s model and unscaled reference model (Eq. (1)) (van der Krogt et al., 2016; Kainz et al., 2018). In summary, we had two models for each participant which were exactly equivalent except for the femoral geometry and corresponding muscle paths and attachments of muscles.

$$F_{scaled}=F_{generic}*{\left(m_{scaled}/m_{generic}\right)}^{\left(2/3\right)}$$ (1)

All models and the corresponding gait analysis data were used to calculate joint angles, joint moments, muscle forces and joint contact forces using inverse kinematics, inverse dynamics, static optimization by minimizing the sum of squared muscle activations and joint reaction load analyses, respectively. Knee and ankle joint markers were only used for scaling and excluded during inverse kinematics. The remaining markers were weighted equally. Maximum marker errors and root-mean-square errors were accepted if less than 4 cm and 2 cm, respectively, as recommended by OpenSim’s best practice recommendations (Hicks et al., 2015). Additional analyses were performed to identify muscle attachments on the femur and obtain the effective directions of muscle forces (van Arkel et al., 2013). The mean waveform of the resultant HCF from all trials was calculated and the trial with the lowest root mean square difference to the mean waveform was selected as a representative loading condition. Similar to previous studies (Yadav et al., 2016; Kainz et al., 2020) nine load instances were selected based on the HCF peaks and the valley in-between during the stance phase. The HCF and muscle forces acting on the femur during the nine load instances were used as loading conditions for FE analysis.

### 2.4 Creation of hexahedral mesh

**FIGURE 1:**
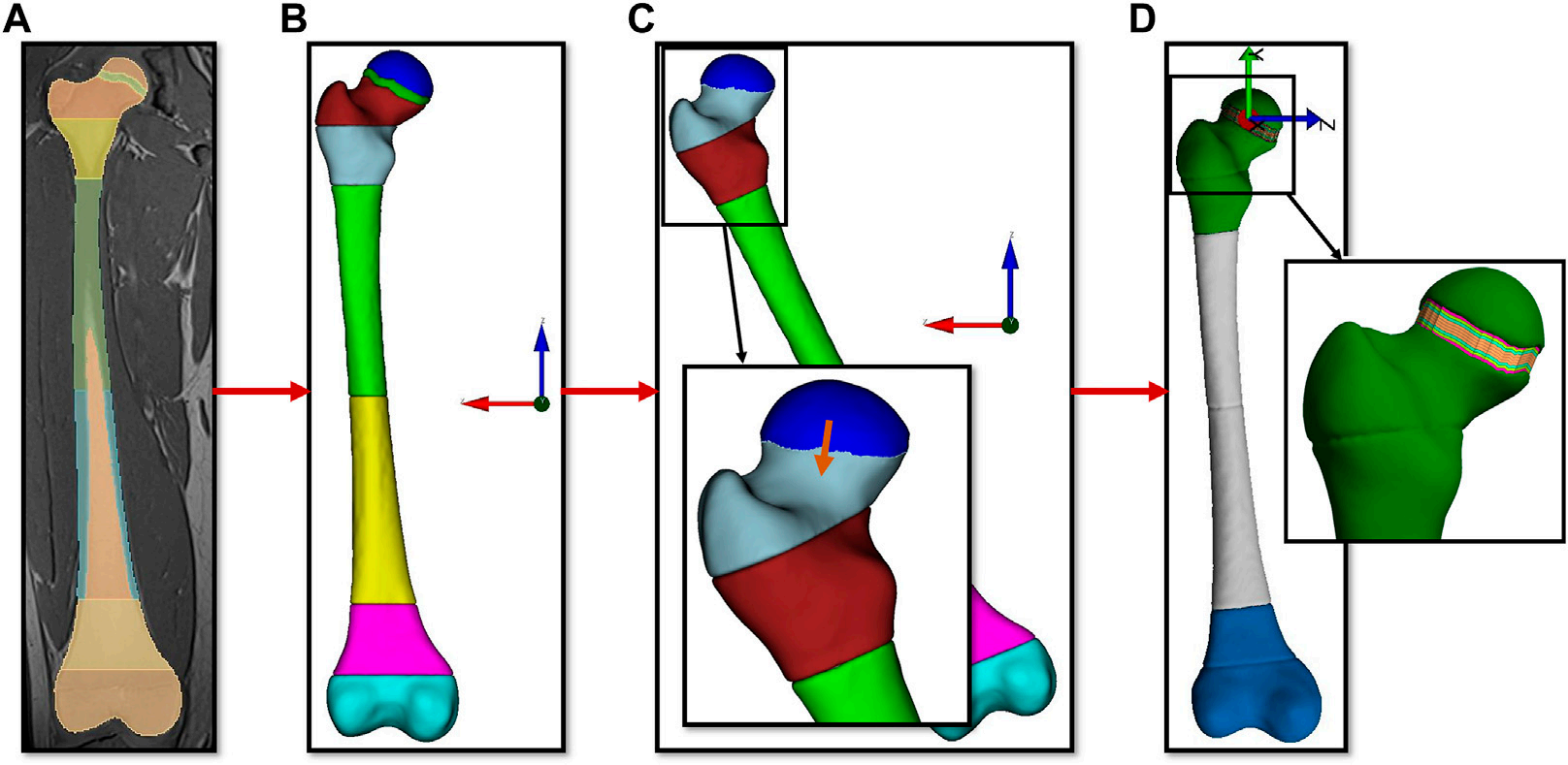
Visual description of the steps performed by the semi-automated toolbox with **(A)** the segmentation of the MRI data as input. Subfigure **(B)** shows the 3D geometry of the femur in the MRI reference system. In subfigure **(C)** the geometry was rotated to have the growth plate parallel to the XY plane, the growth plate was removed, the resulting gap closed by moving the proximal part of the femoral head and the edges smoothed. Sculpt tool of Coreform Cubit was used to create a hexahedral mesh of this geometry. Subfigure **(D)** shows the final mesh with ten laysers of elements aligned with the growth plate.

The mechanobiological model requires hexahedral elements aligned within the growth plate stacked in several layers to define transition zones and enable progressive growth simulations for each layer of the growth plate. We developed the GP-Tool to automatically create hexahedral meshes based on the subject-specific femoral geometry. The STL-files obtained from the segmented femurs were used as input for the GP-Tool. A visual overview of the steps to create a mesh with the GP-Tool is shown in Figure 1.

Using MATLAB’s (MathWorks, Natick, MA, United States) principal component analysis (“pca”-function) the main orientation of the growth plate was determined. All parts of the femur were rotated so that growth plates’ main orientation was parallel to the XY plane. The growth plate itself was removed and the part above the growth plate of the proximal trabecular bone was positioned on the part which is below the growth plate. Smoothing of the intersection region was performed with MeshLab (Cignoni et al., 2008). These steps were necessary to create a continuous mesh to add perfectly aligned hexahedral elements in the growth plate later. Sculpt tool of Coreform Cubit (Coreform, Utah, United States) was used to create a hexahedral mesh with an element size of approximately 1.5 mm. A mesh convergence study was conducted based on three femurs to ensure that the results are not influenced by the number of elements in the mesh (see Supplementary Material). The part above the growth plate was moved to its original position and ten layers with equal height were added and presented the growth plate. Finally, the mesh was optimized to no longer include elements with negative Jacobians. This procedure was performed for each femur of all participants (N = 50) and resulted in meshes of approximately 150.000 nodes and 140.000 elements varying due to different femur sizes.

### 2.5 Finite element analysis

A FE model was created for each femur based on the subject-specific hexahedral mesh and the loading conditions obtained from the MSK simulations. All models were fixed at the femoral epicondyles and HCF and muscle forces were applied as nodal forces for the nine load instances.

For each load instance the HCF was distributed to the closest 100 surface nodes (approximately 2.25 cm^2^) in the direction of the HCF orientation. For each muscle a node was identified which was the closest node to the muscle attachment obtained from the OpenSim simulations (van Arkel et al., 2013). Due to discrepancies in the geometry, e.g., bending of the shaft, between OpenSim’s femur and the participants’ femur derived from MRI an algorithm was used to ensure that the defined muscle attachment was on the same side (anterior/posterior or medial/lateral) of the femur. Nodal forces were applied to this node in x/y/z directions according to the muscle directions obtained from the additional muscle analysis (van Arkel et al., 2013) in order that the resulting force was equal to the muscle force estimated by the static optimization algorithm.

The FE model was duplicated and two different sets of linear elastic materials with Young’s modulus and Poisson ratio described in Table 1 were assigned to the different parts of the femur. The chosen values for material properties were based on literature and previously used values in mechanobiological growth studies (Linde et al., 1985; Rho et al., 1993; Carriero et al., 2011; Yadav et al., 2016; Kainz et al., 2020). A transition zone of three layers (out of the ten layers within the growth plate) between trabecular bone and the growth plate was modeled with linearly decreasing Young’s modulus from the trabecular bone to the growth plate to represent the mineralizing bone tissue (Kainz et al., 2020). FEBio 3 (Maas et al., 2012) was used for FE simulations and to calculate principal stresses.

**TABLE 1:** Sets of material properties used for the different sections of the femur.

Anatomical structure	“Youngs’ modulus”		“Poissons’ ratio”
	“hard”	“soft”	
growth plate	1000	100	0.49
prox. trabecular	10000	2000	0.3
dist. trabecular	10000	5000	0.3
cortical bone	20000	20000	0.3
bone marrow	1	1	0.3

### 2.6 Osteogenic index calculation

The growth rate due to mechanical loading was estimated as the OI (Stevens et al., 1999). Considering that bone mineralizes on the diaphyseal side, the OI was determined for each element of the most distal layer within the growth plate (excluding transition zone). Similar to previous studies (Shefelbine and Carter, 2004; Carriero et al., 2011; Yadav et al., 2016; Yadav et al., 2017; Kainz et al., 2020; Kainz et al., 2021a; Yadav et al., 2021) the OI was estimated using the obtained principal stresses (*σ*_*1*_ *, σ*_*2*_ *, σ*_*3*_) from the FE analysis (Eq. (2)). Hydrostatic stresses ( $\sigma_{H_{i}}$ ) and octahedral shear stresses ( $\sigma_{S_{i}}$ ) were calculated for each load instance (*i = 1…9*) (Equations (3) and (4) and constants *a* and *b* were chosen with values of 0.02 MPa^−1^month^−1^ and 0.01 MPa^−1^month^−1^, respectively, to have a ratio b/a = 0.5 similar to previous studies) (Shefelbine and Carter, 2004; Carriero et al., 2011; Yadav et al., 2016; Yadav et al., 2017; Yadav et al., 2021). The unit of the OI is therefore *month* ^*−1*^. Outliers identified as OI values that were more than three scaled median absolute deviations away from the median (MATLAB’s “isoutlier” function) were replaced by the corresponding minimum or maximum values. These identified outliers were located at the outer edges of the growth plate, which might be due to the lower quality of the hexahedral elements in this region compared to the rest of the growth plate. Therefore, this step was necessary to ensure smoother OI distribution for subsequent comparisons.

$$OI=a*\;\max\left(\sigma_{S_{i}}\right)+b*\;\min\left(\sigma_{H_{i}}\right)$$ (2)

$$\sigma_{S_{i}}=\frac{\sqrt{{\left(\sigma_{1}-\sigma_{2}\right)}^{2}+{\left(\sigma_{2}-\sigma_{3}\right)}^{2}+{\left(\sigma_{3}-\sigma_{1}\right)}^{2}}}{3}$$ (3)

$$\sigma_{H_{i}}=\frac{\sigma_{1}+\sigma_{2}+\sigma_{3}}{3}$$ (4)

### 2.7 Quantification of the osteogenic index

**FIGURE 2:**
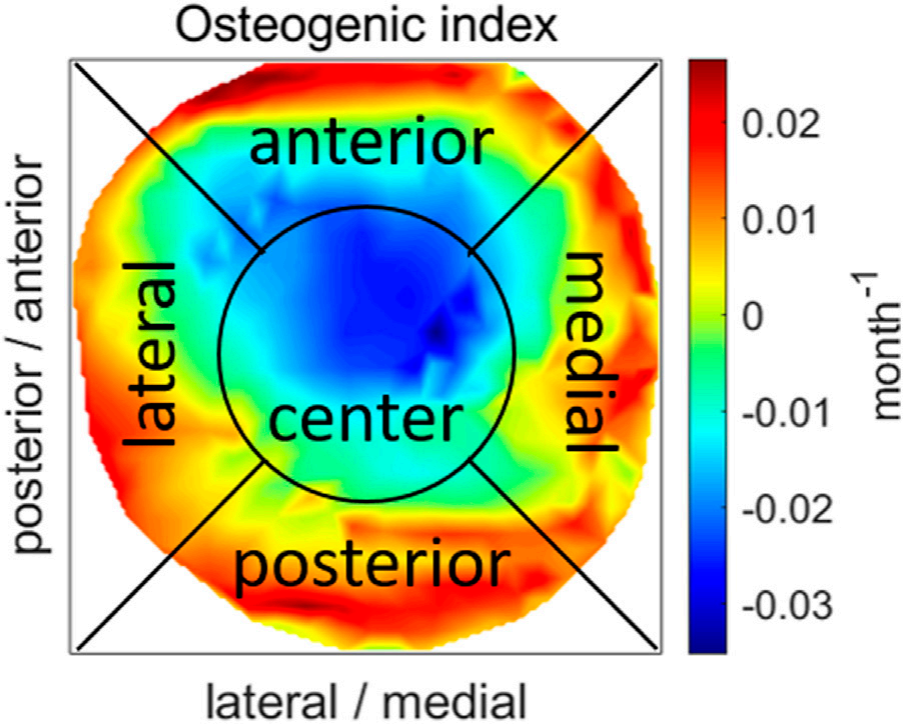
An example of a projection of OI values on the transverse plane showing the division into five regions. The OI distribution is represented using a blue to red color scheme representing low and high values, respectively.

To enable a comparison of OI between growth plates with different shapes, the OI values were projected on the transverse plane according to the elements’ locations and interpolated to a squared grid. A blue to red color scheme was used to visualize OI values representing minimum to maximum OI values, respectively. This resulted in heatmaps of equal size for all growth plates revealing the OI distribution (anterior/posterior and medial/lateral) based on the mechanical loading condition. Subsequently, the squared grid was divided into five regions representing the center, anterior, posterior, lateral and medial sections of the growth plate. The center was defined as a circle with a diameter of 50% of the growth plate’s width and the other regions according to their corresponding anatomical sides of the growth plate separated by the square’s diagonals (Figure 2). Consecutively, the region with the highest and lowest mean value was identified which indicates the area with maximally promoted and inhibited growth, respectively. Since these heatmaps were scaled to each analysis’ minimum and maximum values the magnitude of the OI was neglected. Therefore, we additionally evaluated the range of the magnitude and the mean and median value of the OI.

### 2.8 Osteogenic index reference dataset

A representative reference OI distribution heatmap was created by calculating mean OI values across participants for the TD and the CP group. One can imagine this as stacking up all heatmaps. Due to the different shapes of the growth plates, on the outer edges only few OI values were available. To avoid incorrect values at these locations, mean values were only calculated for locations if data of more than half of the femurs, i.e. more than 12 and 13 for CP and TD, respectively, were available.

### 2.9 Intra-subject variability

Two main metrics were used to quantify the intra-subject variability of the OI for each participant. Firstly, the regions with the highest and lowest mean value were compared between each participant’s left and right femur. Secondly, to quantify differences in shape and distribution of the OI (e.g. ring shape vs. linear-gradient), an image comparison between the heatmaps of the left and right femur was performed using OpenCV’s template matching (Bradski, 2000). The “TM_CCOEFF_NORMED”-method was used which computes one resultant value between 0 and 1 that represents the probability that images are equal. For a more comprehensible metric, the result was subtracted from 1 so that high values indicate high variability. Additionally, to account for the magnitude of the OI, the difference of the mean values and difference of the ranges between the left and right OIs were calculated. Subsequently, independent t-tests were performed to identify differences of the intra-subject variability between the CP and TD group.

### 2.10 Inter-subject variability

To assess the inter-subject variability between groups, we analyzed the mean occurring OI values in each region. Firstly, the mean value of each region was normalized to the range of the individual OI. Then, these normalized values for each region were compared between CP and TD group with independent t-tests to evaluate differences in general OI distribution. To identify statistically significant differences in the variability, the standard deviations of each region were compared between groups with a Levene’s test.

Additionally, we compared each OI heatmap with all OI heatmaps of the same group, i.e. each OI of a TD child was compared to all other OIs of TD children, with the before mentioned image comparison method. Comparisons of equal heatmaps (e.g. “TD01 left side” vs. “TD01 left side”) were neglected. Subsequently, the mean variability to other OIs of the group was calculated. This measure indicates the difference of shape and distribution of a single OI within its group. Next, the inter-subject variability within each group was compared between TD children and children with CP. Again, to account for the magnitude of the OI, the mean values and the ranges of the OIs were compared between groups with a two-sampled t-test.

### 2.11 Influence of MSK model and FE material properties

The sum of all muscle forces acting on the femur as well as the magnitude of the HCF at the first and the second peak were compared between the MSK simulations with generic-scaled and personalized models. Within each group (TD and CP), all parameters were tested for significant differences with two-sampled t-tests. Between groups, muscle forces and HCF of the personalized MSK model were compared with independent t-tests.

To answer our technical research questions on the influence of the MSK model and the material properties on the OI, we performed the same comparisons and statistical tests as described above for the simulation results obtained with altered loading conditions and altered material properties, separately. Subsequently, we identified differences in outcomes due to different loading conditions and material properties. Additionally, we evaluated changes of the regions with the highest mean value compared to the base analysis with “hard” material properties and personalized MSK models.

### 2.12 Geometrical analysis

To identify possible reasons for observed differences of the OI between the CP and the TD group, the shape, the location and the orientation of the growth plates were analyzed. The shape was quantified by fitting a sphere through nodes in the middle of the growth plate. If the sphere’s midpoint was distal of the growth plate the shape was convex, otherwise it was concave. The radii of these spheres were compared between groups. The location of the growth plate was determined by calculating the distance between the intersection point of the neck axis and the femur’s proximal surface and the mean location of all nodes within the growth plate. The neck length was quantified as the femoral head radius plus the distance between the femoral head center and the point where neck and shaft axis have the minimal distance to each other. The location of the growth plate was normalized to the length of the femoral neck. To quantify the orientation of the growth plate the angles between the normal vector to the growth plate (obtained during mesh creation) and 1) the femur coordinate system and 2) the orientation of the mean and maximum HCF during stance phase in the anatomical planes were calculated. Additionally, angles between the neck axis and 1) the orientation of the mean and maximum HCF in the anatomical planes and 2) the normal vector to the growth plate were computed.

## 3 Results

### 3.1 Participant exclusion and FE analysis metrics

In total, 6 out of 50 (12%) FE analysis with “hard” material properties and the personalized MSK model did not converge and therefore four participants (all children with CP; in two participants, left and right side did not converge) had to be excluded from our study because intra-subject comparisons were impossible. Data of these participants was not used in any analysis. Therefore, all presented results include data of 8 children with CP and 13 TD children, i.e. 42 femurs in total. In the analysis sets with the generic-scaled MSK model and “soft” material properties 2 (4.75%) and 4 (9.5%) out of the remaining 42 FE analysis did not converge, respectively.

### 3.2 Osteogenic index reference dataset

**FIGURE 3:**
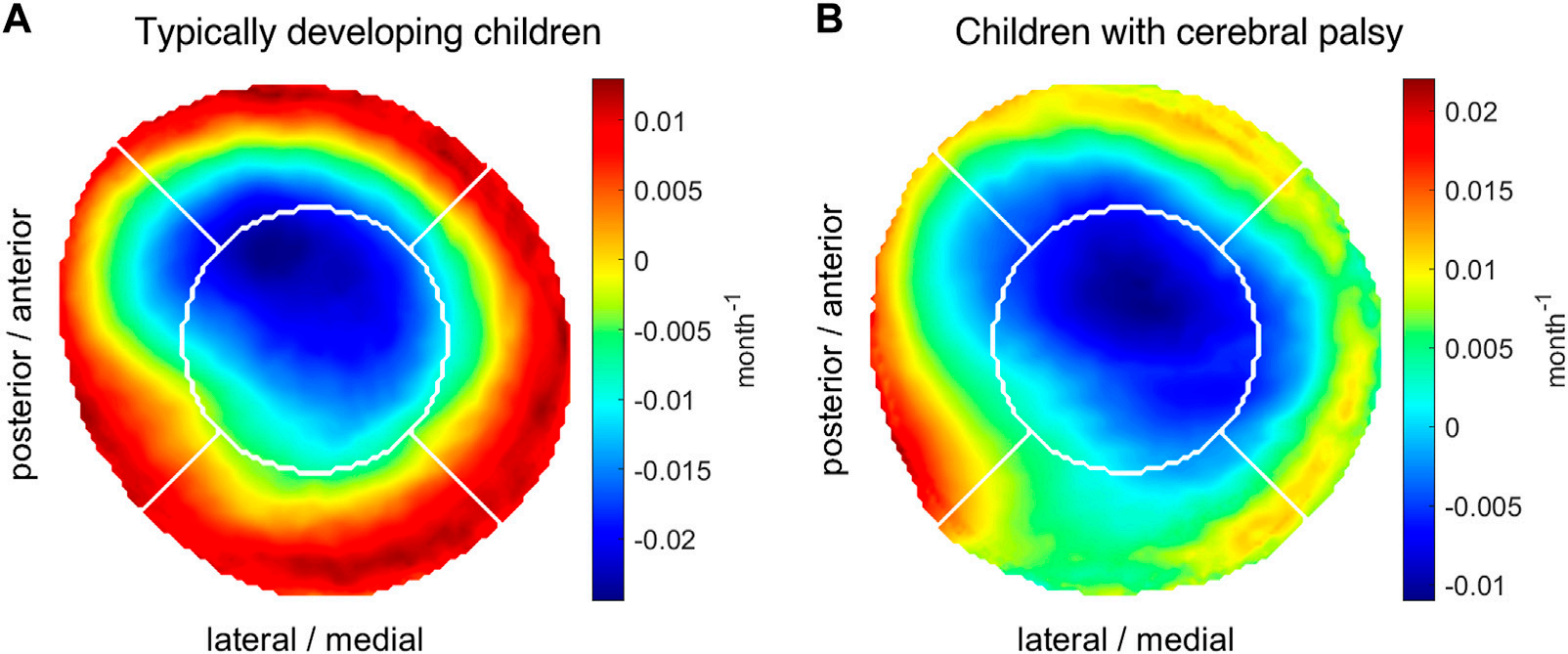
Average growth plate shape and OI distribution in TD children **(A)** and children with CP **(B)**. The OI distribution is represented using a blue to red color scheme representing low to high values, respectively.

The representative reference OI distribution heatmap generated from data of 26 TD children’s femurs showed a ring shape with low values in the center region and high values at the border of the growth plate (Figure 3A). The descending order of the regions according to their highest mean was posterior, medial, lateral, anterior and center. The reference OI for TD children was distributed between −0.024 months^−1^ and 0.013 months^−1^ with a mean value of −0.003 months^−1^.

The reference OI distribution generated from data of 16 femurs of children with CP had a different shape with highest values on the lateral side of the growth plate (Figure 3B). An indication of a ring shape distribution, similar to the TD children, was observed. The regions with highest mean values in descending order were lateral, posterior, anterior and medial followed by the center. The OI values ranged from −0.011 months^−1^ to 0.022 months^−1^ with a mean of 0.003 months^−1^.

### 3.3 Intra-subject variability

**FIGURE 4:**
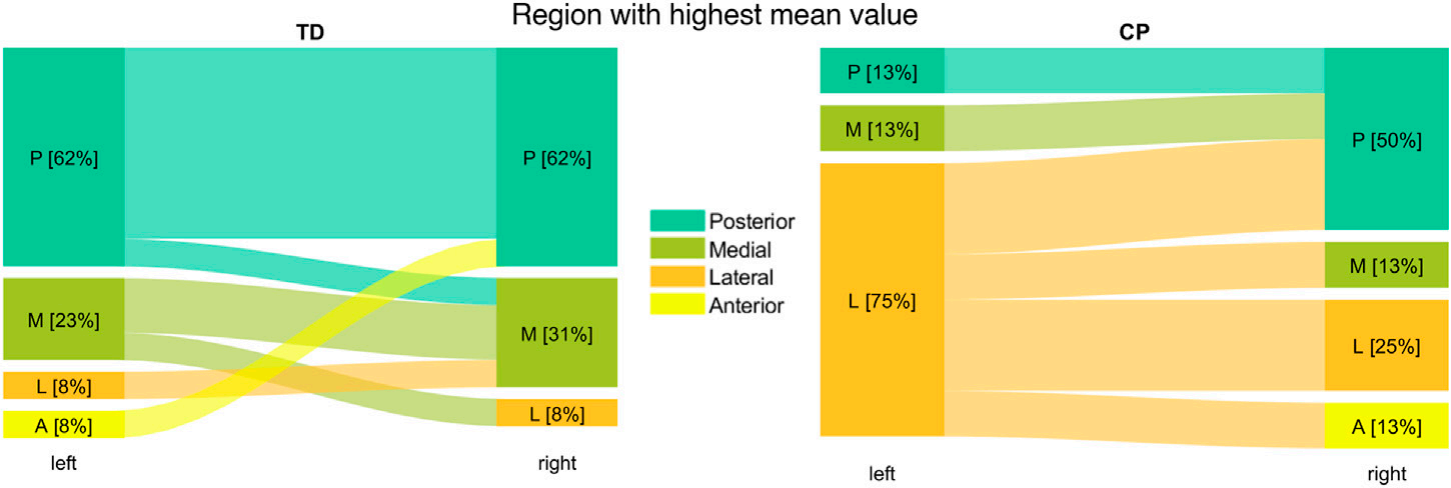
Intra-subject comparison of region with highest mean OI value between left and right growth plate for TD children (left) and children with CP (right).

When comparing the left with the right growth plate of each participant, the highest mean OI value occurred at different regions in 30.8% (n = 4) of TD children and in 62.5% (n = 5) of children with CP (Figure 4). The lowest mean OI value was observed at the center region in all TD children and most children with CP. In one child with CP the lowest mean OI value occurred in the medial region and in another child with CP in the posterior region.

**FIGURE 5:**
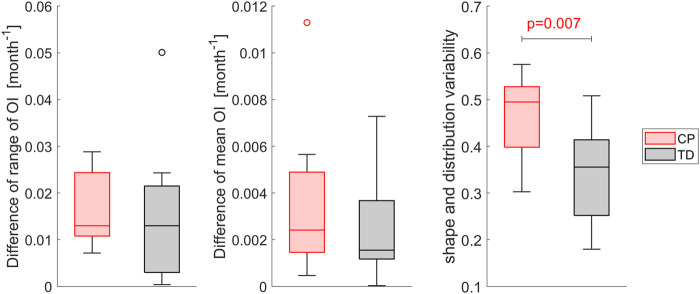
Comparison of the intra-subject variability of the magnitude of OI, i.e. mean value and range, and the variability assessed with template matching of the heatmaps of the left and right OI between children with CP and TD children. Higher variability values indicate a higher difference in shape and distribution, a value of 0 indicates equal OI shape and distribution. Significant differences were quantified with independent t-tests.

The differences of the mean OI value as well as the range of OI values between the left and right side were not significantly different between both groups. The image comparison of the heatmaps to quantify the variability of the shape and OI distribution between left and right growth plate showed a significantly higher (*p* < 0.01) intra-subject variability in children with CP compared to TD children (Figure 5).

### 3.4 Inter-subject variability

**FIGURE 6:**
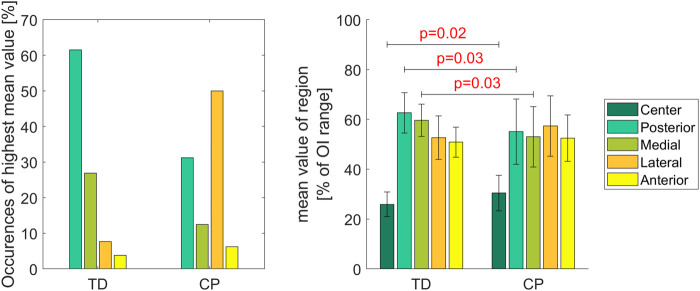
The left diagram shows how often the highest mean value was observed in a specific region. In the right diagram the colored bars represent the mean value of each region normalized to the range of the individual OI. Significant differences were quantified with independent t-tests. Vertical error bars indicate the standard deviation between participants within groups.

In 61.5% (n = 16) of the femurs of the TD group the highest mean value of the OI was observed in the posterior region of the growth plate followed by the medial region (27%; n = 7). In children with CP the lateral (50%; n = 8) followed by the posterior (31%; n = 5) region were the areas with the most occurred highest mean OI values (Figure 6 left).

The mean values of the posterior and medial regions were significantly higher in TD children compared to children with CP whereas significantly lower values were observed in the center region for the TD children. Levene’s test showed significant differences of the variances (*p* < 0.05) in the posterior and medial region between groups indicating that in children with CP the variability is higher compared to TD children (Figure 6 right).

**FIGURE 7:**
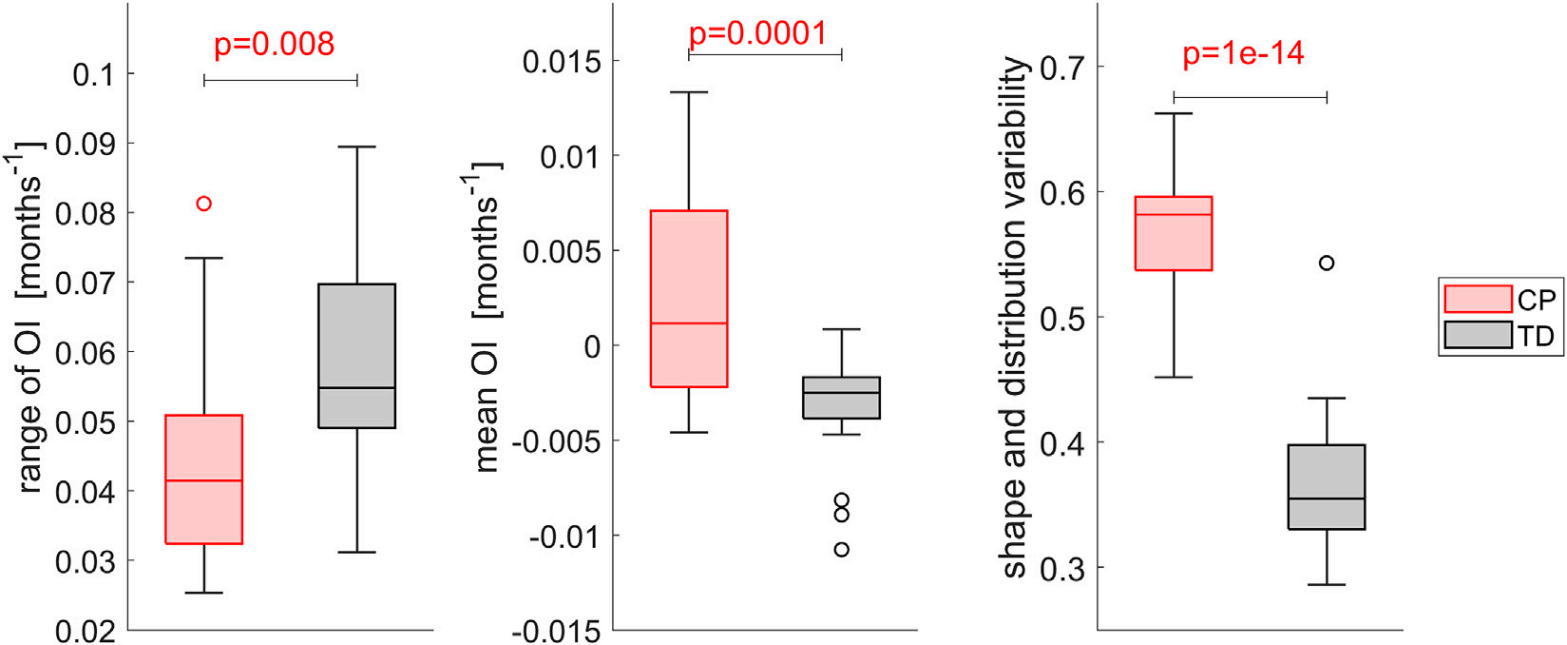
Comparison of the inter-subject variability of the magnitude of OI, i.e. mean value and range, and the variability assessed with template matching of the heatmaps. Each OI was matched with all other OIs of its group and the mean variability compared between groups. Higher variability values indicate a higher difference in shape and distribution, a value of 0 indicates equality. Significant differences were quantified with independent t-tests.

The range of the OI as well as the mean OI differed significantly (*p* < 0.01) between the TD and CP group. In TD children, the range of the OI was higher and lower mean values were observed. Comparing each OI heatmap with all other OI heatmaps of its group revealed significantly higher (*p* < 0.001) inter-subject variability in the CP compared to the TD group (Figure 7).

### 3.5 Geometrical analysis

**FIGURE 8:**
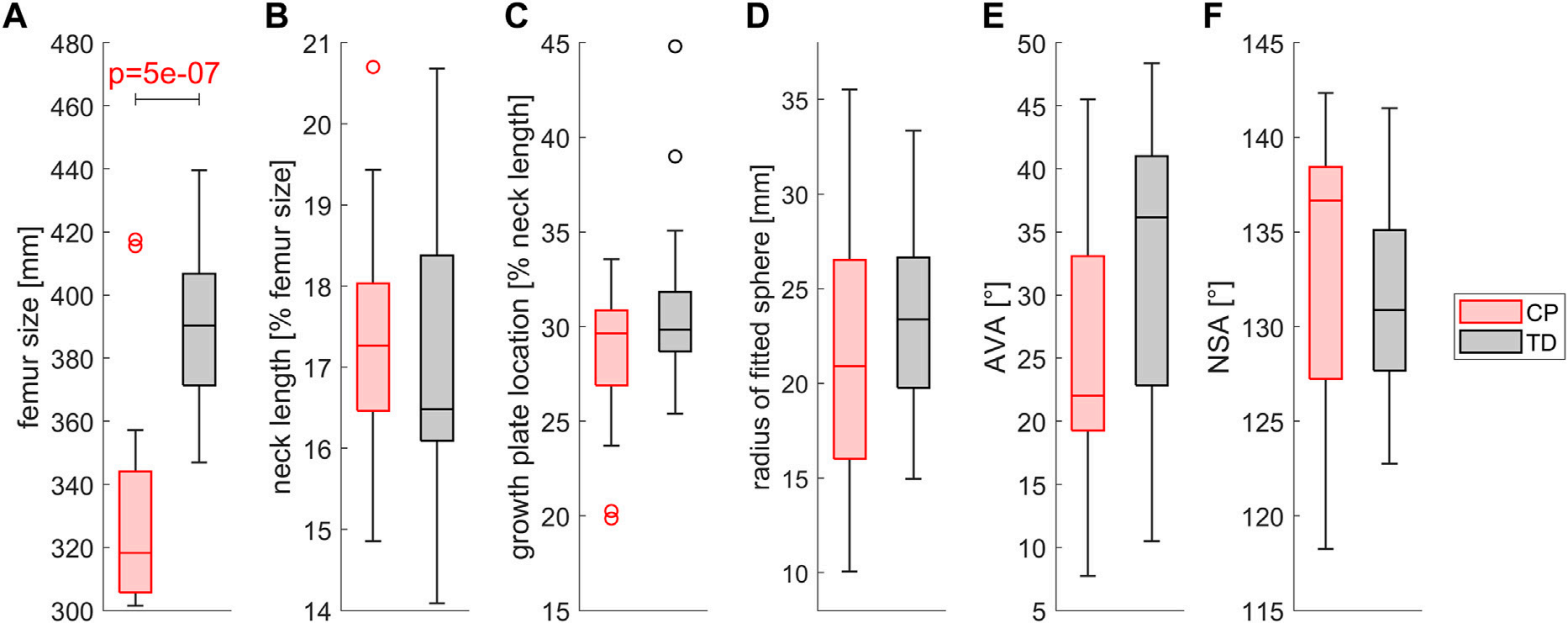
Comparison of **(A)** the femur size, **(B)** the neck length, **(C)** the growth plate location, **(D)** the shape of the growth plate, **(E)** the AVA and **(F)** the NSA between children with CP and TD children. Growth plate location was assessed as the distance between the intersection point of the neck axis and the proximal surface and the middle of the growth plate. The shape of the growth plate was assessed by fitting a sphere into the nodes of the middle layer of the growth plate. Significant differences were quantified with independent t-tests.

The femurs of TD children were significantly (*p* < 0.001) larger compared to those of children with CP. No significant differences were observed for the neck length and the growth plate location between groups normalized to the femur’s length and its neck, respectively. A wide range of AVA and NSA was present in the analyzed dataset but without any statistical differences between the CP and TD group. The shape of the growth plate did not differ between groups, all had a convex shape with radii between 10mm and 35 mm (Figure 8).

**FIGURE 9:**
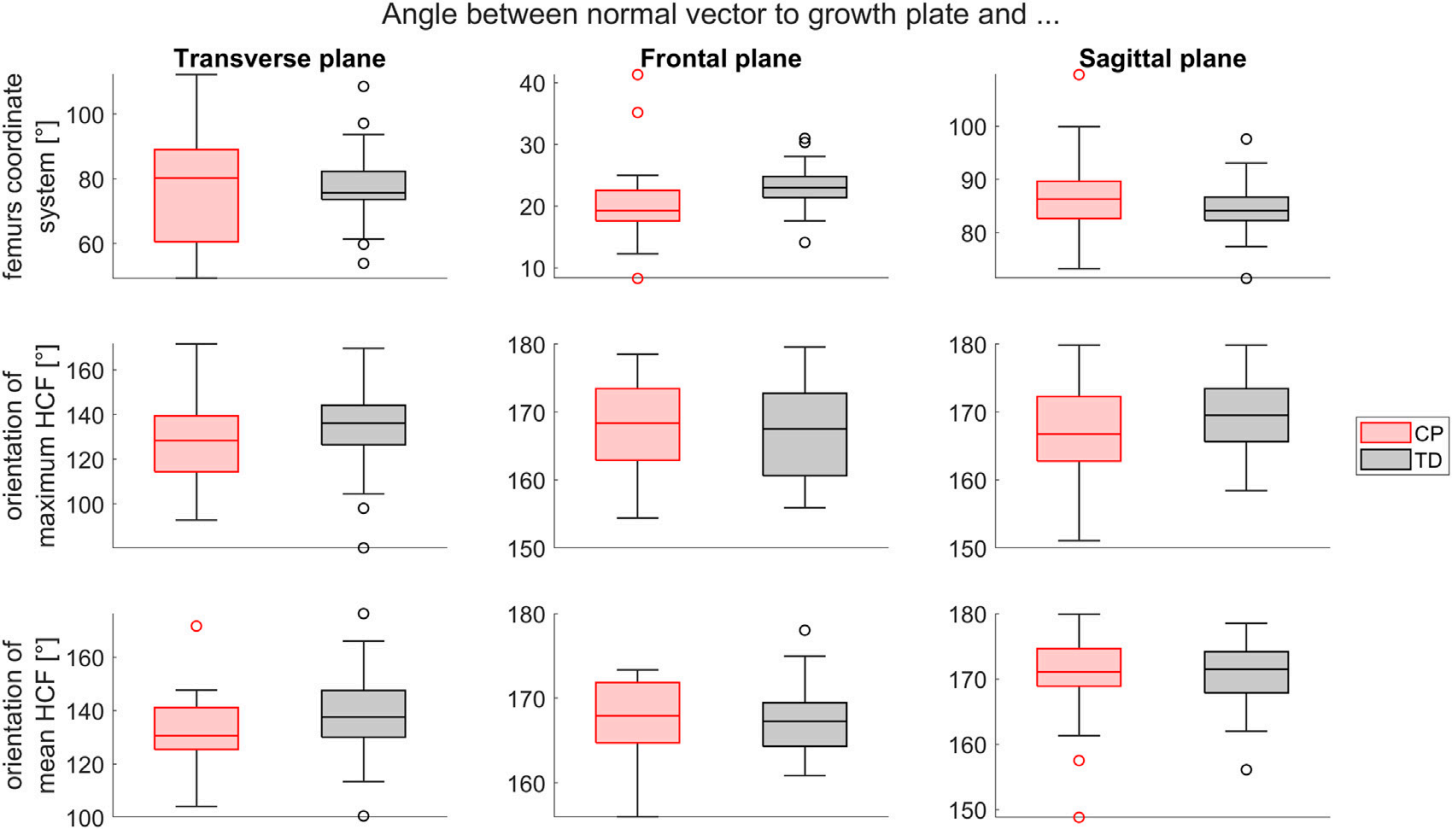
Comparison of the growth plate orientation (normal vector) between the CP and TD group in relation to the femur’s coordinate system (top), the maximum HCF (middle) and the mean HCF during stance phase (bottom) in transverse, frontal and sagittal plane. Significant differences were quantified with independent t-tests.

The orientation of the growth plate was similar in relation to the femur’s coordinate system between children with CP and TD children. The angles between the orientation of the maximum and mean occurring HCF during stance phase and the vector normal to the growth plate did not differ significantly between groups (Figure 9).

**FIGURE 10:**
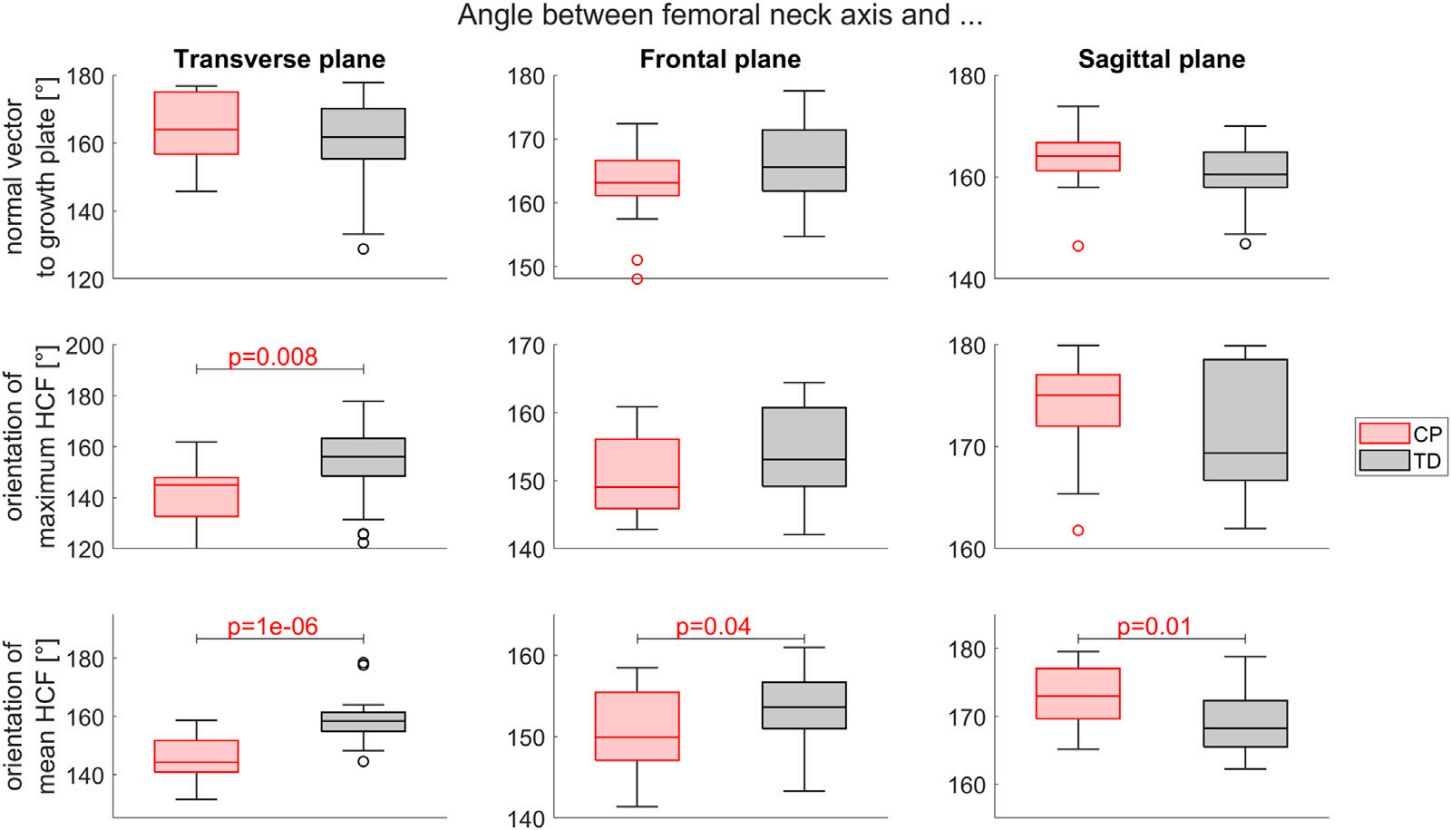
Comparison of the neck axis orientation between the CP and TD group in relation to the growth plates normal vector (top), the orientation of the maximum HCF (middle) and the mean HCF during stance phase (bottom) in transverse, frontal and sagittal plane. Significant differences were quantified with independent t-tests.

No significant difference was observed between the CP and TD group for the angle between the neck axis and the normal vector to the growth plate. The angle between the femoral neck axis and the orientation of the maximum (*p* < 0.05) and mean (*p* < 0.001) HCF was significantly different between CP and TD children in transverse plane. A statistically significant difference between CP and TD children was also observed in the frontal and sagittal plane for the angle between the neck axis and mean HCF during stance phase (Figure 10).

### 3.6 Influence of MSK model

**FIGURE 11:**
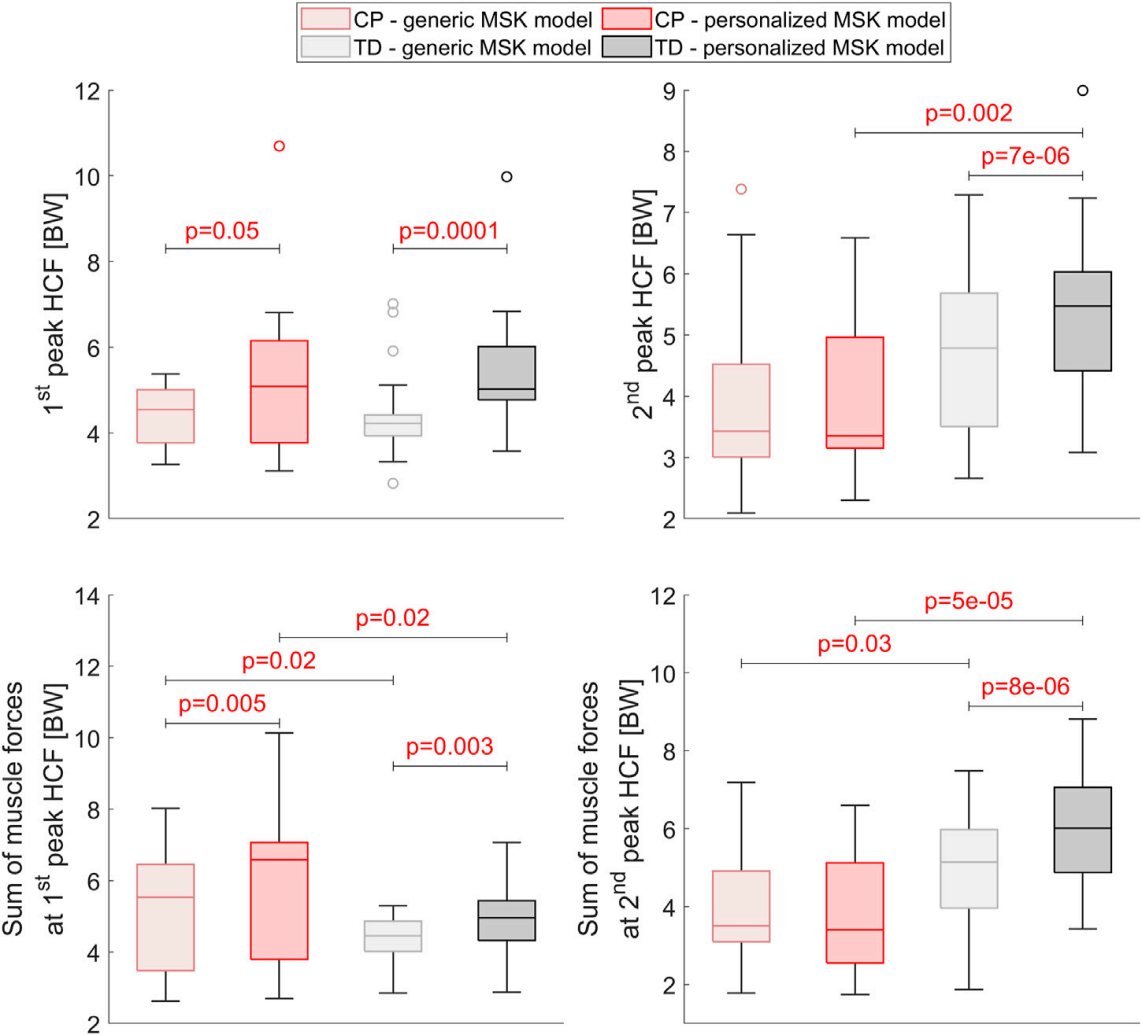
Comparison of the HCF and the muscle forces acting on the participants’ femurs between the generic-scaled and the personalized MSK model within the CP and TD group as well as between groups for the personalized MSK model. Significant differences were quantified with dependent and independent t-tests within groups and between groups, respectively.

The use of a generic-scaled instead of personalized MSK model changed the loading condition, i.e. HCF and muscle forces on the participants’ femurs (Figure 11). At the first peak of the HCF, the magnitude of the HCF and the sum of all muscles acting on the femur were significantly lower in both groups when using a generic-scaled model. In TD participants, the magnitude of the HCF and the muscle forces at the second peak of HCF were significantly lower with the generic-scaled compared to the personalized model. Independent t-tests showed significantly higher values of HCF peak as well as the sum of all muscle forces acting on the femur at both HCF peaks for TD compared to CP children. Two out of the 42 FE analysis did not converge when using the generic-scaled loading conditions. Against the general observation that HCF and muscle forces were lower when using a generic-scaled MSK model, in these two analyses the maximum HCF magnitude increased which led to non-convergence in the FE analysis.

**FIGURE 12:**
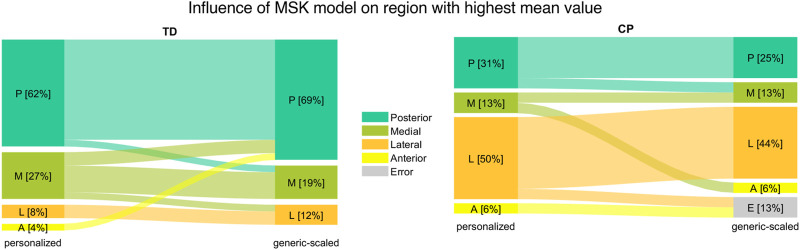
Comparison of region with highest mean value between the generic-scaled and personalized MSK model for TD children (left) and children with CP (right).

The region with the highest mean OI value changed due to the MSK model that was used in 2 (14.3%) and 5 (19.2%) participants of the CP and TD group, respectively (Figure 12). The intra-subject comparison between left and right OI showed that in 30.8% (n = 4) of TD children and in 71.4% (n = 5 out of 7) of children with CP the highest mean OI value occurred in different regions of the growth plate. No statistically significant changes were observed for the differences of the mean OI, the range of the OI and the OI variability between the left and right side due to the MSK model that was used. The range of OI did not significantly differ between the CP and TD group when using the loads from the generic-scaled MSK models, whereas it was significantly different with personalized MSK models. Additional plots, similar to Figure 5; Figure 6 but based on the generic-scaled MSK model can be found in the Supplementary Material.

### 3.7 Influence of FE material properties

**FIGURE 13:**
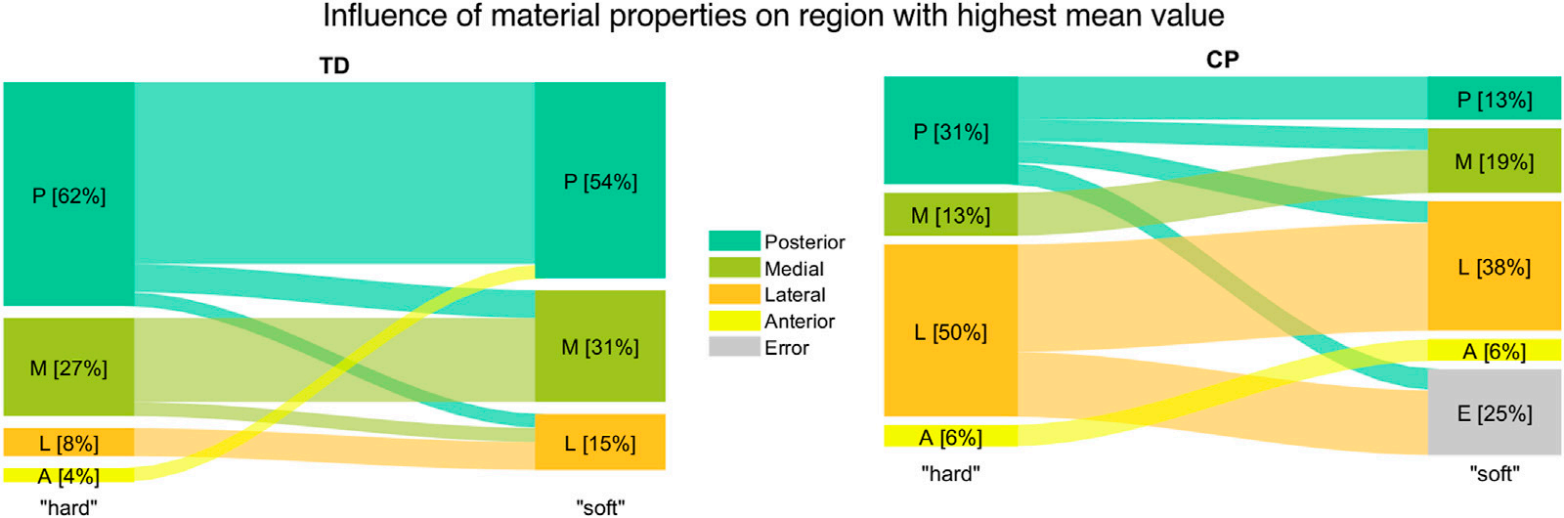
Comparison of region with highest mean value between FE analysis with “hard” and “soft” material properties for TD children (left) and children with CP (right).

For four children with CP the FE analysis did not converge with the “soft” material properties. This reduced the number of intra-subject comparisons for CP children to 5 participants. The region with the highest mean OI value changed due to the material properties in 2 (16.7%) and 5 (19.2%) participants of the CP and TD group, respectively (Figure 13). The intra-subject comparison between left and right OI showed that in 38.5% (n = 4) of TD children and in 60% (n = 3 out of 5) of children with CP the highest mean OI value occurs in different regions of the growth plate. With “soft” material properties, there was no significant difference of the intra-subject variability of the shape and distribution between the left and right side. Inter-subject comparison between the CP and TD group with “soft” material properties showed no significant differences in mean values for all regions. The mean OI was not significantly different between groups whereas this was the case for “hard” material properties. Additional plots, similar to Figure 5; Figure 6 but based on the “soft” material can be found in the Supplementary Material.

## 4 Discussion

The aim of this study was to quantify and compare the OI in the proximal growth plate of the femur in a comparably large cohort of TD children and children with CP with subject-specific loading, femoral morphology as well as growth plate shape and orientation. The GP-Tool based on freely available software was developed, which enables one to create subject-specific FE models and perform the multi-scale simulations in a simple and comprehensive way. The GP-Tool has been shared with the community (https://github.com/WilliKoller/GP-Tool) to enable peers to conduct mechanobiological growth studies with larger sample sizes and enhance our understanding of femoral bone growth. In this study, the analyzed dataset included 26 femurs of TD children and 16 femurs of children with CP and empowered us to create a reference OI as well as to evaluate the intra- and inter-subject variability. The within-group variability in range of OI, mean OI, OI shape and distribution were significantly different between groups, suggesting different bone growth patterns may be present in CP and TD children.

The reference OI for TD children showed a clear ring shape with high values on the outside and low values in the center which indicates more growth on the outside compared to the inner part of the growth plate. This could support the finding that with time and under normal loading conditions, the growth plate changes its shape from a spherical to a flat disc (Kandzierski et al., 2012).

In children with CP the reference OI clearly differed from those of TD children with the maximum values occurring in the lateral region. Considering the high variability of femoral morphology and femoral loading patterns in children with CP, the presented reference OI does not reflect the whole population of children with CP and therefore should be interpreted with caution. Some children with CP had OIs that were similar to the reference OI of TD children while others were very different.

The inter-subject analyses showed that in most femurs of TD children, the region with the highest mean OI values were the posterior followed by the medial region. In both regions, we found significantly higher OI values within the TD compared to the CP group. Most previous studies based on loading conditions estimated from gait of TD children also found a ring-shaped OI distribution with the highest values on the posterior side of the growth plate (Yadav et al., 2017; Kainz et al., 2020). Carriero et al. (2011) reported a linear-gradient OI distribution with the highest values on the lateral side for a healthy child, which was contrary to the OI of our TD children but similar to the OI of our participants with CP. Carriero et al. (2011) however, conducted their simulations based on a model with a simplified growth plate, i.e. flat disc. The low participant number and simplified growth plate (Yadav et al., 2016) might explain the contrary results from Carriero et al. (2011) compared to the more recent investigations.

The analysis of the intra-subject variability showed that in most TD children the region with the highest mean value was equal between the left and right side while in the CP group this was only the case for approximately a third of the participants. The variability of the shape and distribution of the OI also revealed a significantly higher intra-subject variability in children with CP compared to TD children which could lead to asymmetric femoral growth.

In the analyzed dataset, the femurs of children with CP were significantly smaller compared to the TD group but the morphology in terms of neck length, AVA, NSA, growth plate location and growth plate shape was similar between groups. All morphological parameters were within the range of TD values reported in a study based on 508 participants (Szuper et al., 2015).

The use of generic-scaled instead of image-based MSK models significantly changed the femoral loading conditions, i.e. muscle forces and HCF, but the general findings remained similar. The only statistical differences between analysis based on the generic-scaled and personalized MSK model were observed for the range of the OI, whereas the generic-scaled MSK model led to fewer differences between the CP and TD group.

Four analyses did not converge when using material properties with the compliant material, i.e., low Young’s modulus. In linear elastic materials, which were used in this study, lower stiffness leads to larger displacements. When elements are deformed resulting into inverted elements, the FE solver cannot continue the analysis. In theory, the stress within a material only depends on the force and the cross-section of the geometry and therefore, stresses within the growth plate should not be influenced by the used material properties. However, in FE analysis the material properties are used to initially calculate the global stiffness matrix. Then, displacements are estimated and subsequently used to calculate the stresses (Bathe, 2006, 149ff). Hence, changes of material properties have an impact on observed stresses within the growth plate. However, observed changes in our analyses were minimal with the only statistical difference in the mean OI value reducing the difference between CP and TD group.

Four participants had to be excluded because in total, 6 out of 50 FE analysis did not converge with stiff material properties. The reasons for this were mainly tremendously high HCFs (∼4000 N, above 10 times body weight) or very thin cortical bone. Thin cortical bone, i.e., only two or three element layers on the femoral shaft (bone marrow has low Young’s modulus and can be neglected), is unlikely to support the full MSK loading. In future studies, attention has to be paid to the segmentation of the cortical bone. Unrealistic and insufficient cortical thickness and/or unrealistic high HCF might not converge and therefore make the estimation of the OI impossible.

We only estimated growth plate stresses and quantified the OI but did not simulate femoral growth. Femoral growth can be predicted with an additional FE analysis including a method to estimate the growth direction. To do this, different methods have been suggested to define the growth direction, i.e. femoral neck deflection direction or principal stress direction (Yadav et al., 2016). None of these methods have been validated based on longitudinal data and therefore we decided not to include the additional analysis in our study.

In children with CP muscle morphology and motor control are often altered compared to TD children, i.e. shorter muscle belly, muscle spasticity, decreased muscle volume and strength (Hanssen et al., 2021; Peeters et al., 2023). Assessment of individuals’ muscle properties, e.g. maximum isometric muscle forces, with clinical tests is challenging in children with CP (Kainz et al., 2018). We accounted for the individuals’ bony geometry which has been shown to have a larger impact on hip joint contact forces than the personalized motor control (Kainz et al., 2021b) Nevertheless, future studies could include ultrasound measurements to personalize muscle parameters (Schless et al., 2018), include a spasticity model (Falisse et al., 2018; Falisse et al., 2020) and account for the patient-specific motor control (Veerkamp et al., 2019) to further improve the accuracy of the MSK simulations.

Our study included the following limitations. First, bone is a highly complex structure with anisotropic material properties. In this study, the material of the bone was simplified to have linear elastic properties. Previous studies modelled the bone similarly (Shefelbine and Carter, 2004; Carriero et al., 2011; Yadav et al., 2016; Kainz et al., 2020; Yadav et al., 2021). FEBio supports complex materials, therefore, the developed GP-Tool can be modified to include more realistic materials for future studies. Second, during the automated meshing, a small number of elements with low quality metrics (at the very outer border) of the growth plate were removed. This was necessary to avoid inverted elements under load to ensure successful FE analysis. Third, the MSK models included only the personalized femoral morphology whereas the geometry of the tibia was neglected. Changes of bony geometry alters moment arms of muscles and subsequently has an impact on the estimated muscle forces and HCF. However, tibial torsion only affects a limited number of muscles and therefore we assume the impact on HCF is negligible. Studies which investigate the impact of tibial torsion on HCF should be carried out to verify our assumption. Fourth, we used a static optimization algorithm to compute muscle activities. Approaches including electromyography (EMG) data might better represent the individuals’ motor control (Pizzolato et al., 2015). Due to lack of EMG data in all our participants we could not perform EMG-informed analysis and therefore decided to use static optimization. Femoral geometry has a bigger impact on HCF than including EMG data in the optimization (Kainz et al., 2021b) and therefore we would expect to get similar OI with an EMG-informed approach.

The findings of this paper can help to identify possible pathological loading on the growth plate which might result in atypical femoral bone growth. If pathological loading is noticed at an early stage, subject-specific therapies like real-time biofeedback training could be used to alter joint loads (Comellas and Shefelbine, 2022; Uhlrich et al., 2022) and therefore change growth plate stresses. However, further studies have to be carried out to identify how different walking pattern and motor control strategies influence growth plate stresses.

To conclude, we developed the GP-Tool to create subject-specific FE models and estimate the OI in a semi-automatic way. The code of the GP-Tool has been made freely available to the research community as well as an executable file for users without a MATLAB license. On a standard notebook, the complete process of creating a subject-specific mesh from STL files, setting up and running the FE analysis takes around 30 min. We hope the GP-Tool encourages peers to conduct similar studies with large sample sizes to improve our general insights in femoral bone growth and to support clinical decision making in the near future.

## Data Availability

The raw data supporting the conclusions of this article will be made available by the authors, without undue reservation.
