# Supplementary material for: Intra- and inter-subject variability of femoral growth plate stresses in typically developing children and children with cerebral palsy
Source: Front Bioeng Biotechnol. 2023 Feb 24;11:1140527. doi: 10.3389/fbioe.2023.1140527 (PMC9999378; doi:10.3389/fbioe.2023.1140527)
Supplement: Supplementary file 1 [file DataSheet2.pdf]

Supplementary Material

Intra- and inter-subject variability of femoral growth plate stresses in typically developing children and children with cerebral palsy

Willi Koller[*]**, Basílio Gonçalves, Arnold Baca, Hans Kainz**

*** Correspondence:** Corresponding Author: willi.koller@univie.ac.at

1 Influence of MSK model

Data of 7 CP children (i.e. 14 femurs) and 13 TD children (i.e. 26 femurs) were used.

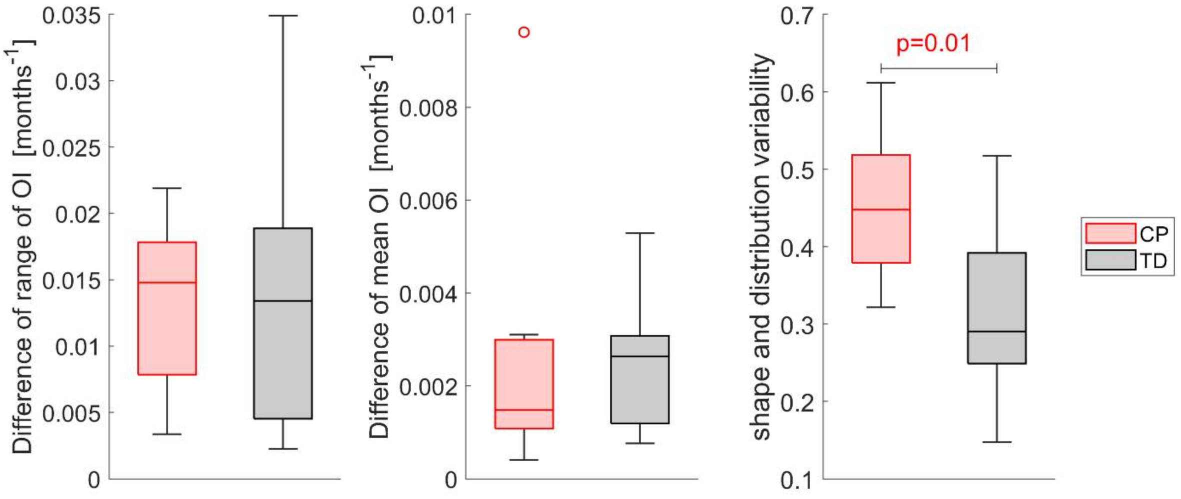

Supplementary Figure 1. Comparison of the intra-subject variability of the magnitude of OI, i.e. mean value and range, and the variability assessed with template matching of the heatmaps of the left and right OI between children with CP and TD children. Higher variability values indicate a higher difference in shape and distribution, a value of 0 indicates equal OI shape and distribution. Significant differences were quantified with independent t-tests.

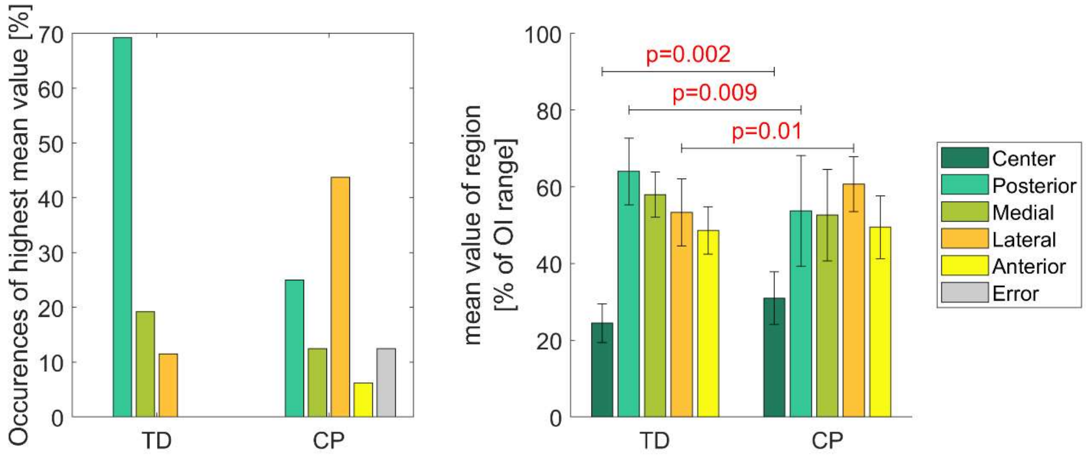

Supplementary Figure 2. The left diagram shows how often the highest mean value was observed in a specific region. In the right diagram the colored bars represent the mean value of each region normalized to the range of the individual OI. Significant differences were quantified with independent t-tests. Vertical error bars indicate the variability between participants within groups.

2 Influence of material properties

Data of 5 CP children (i.e. 10 femurs) and 13 TD children (i.e. 26 femurs) were used.

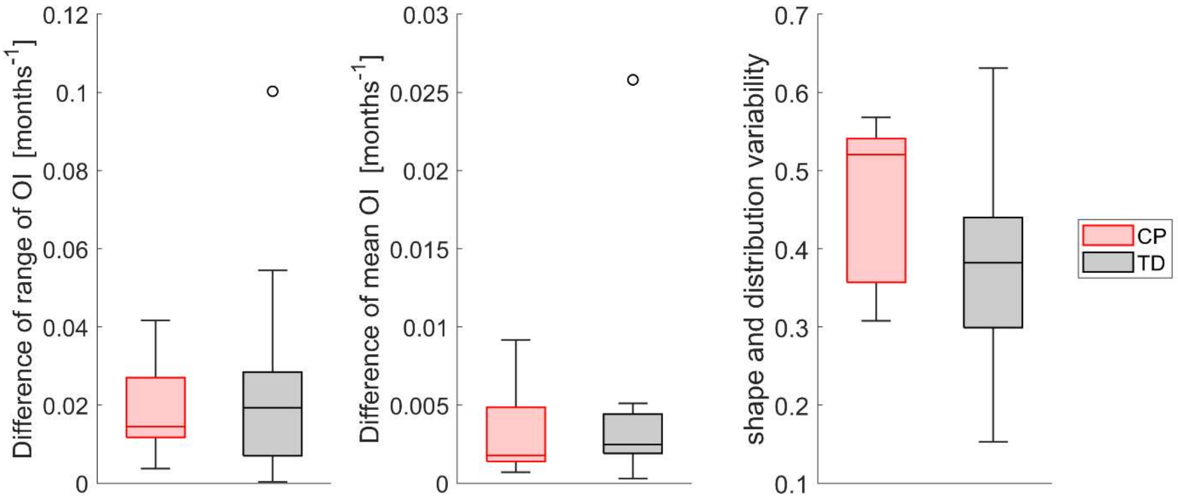

Supplementary Figure 3. Comparison of the intra-subject variability of the magnitude of OI, i.e. mean value and range, and the variability assessed with template matching of the heatmaps of the left and right OI between children with CP and TD children. Higher variability values indicate a higher difference in shape and distribution, a value of 0 indicates equal OI shape and distribution. Significant differences were quantified with independent t-tests.

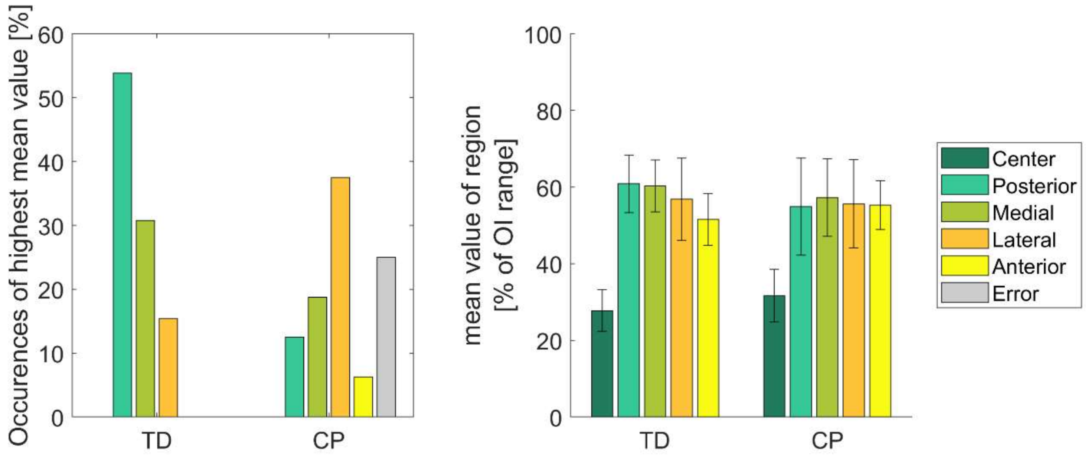

Supplementary Figure 4. The left diagram shows how often the highest mean value was observed in a specific region. In the right diagram the colored bars represent the mean value of each region normalized to the range of the individual OI. Significant differences were quantified with independent t-tests. Vertical error bars indicate the variability between participants within groups.
