# Supplementary material for: Intra- and inter-subject variability of femoral growth plate stresses in typically developing children and children with cerebral palsy
Source: Front Bioeng Biotechnol. 2023 Feb 24;11:1140527. doi: 10.3389/fbioe.2023.1140527 (PMC9999378; doi:10.3389/fbioe.2023.1140527)
Supplement: Supplementary file 2 [file DataSheet1.pdf]

Supplementary Material

Intra- and inter-subject variability of femoral growth plate stresses in typically developing children and children with cerebral palsy

Willi Koller[*], Basílio Gonçalves, Arnold Baca, Hans Kainz

*** Correspondence:** Corresponding Author: willi.koller@univie.ac.at

1 Mesh convergence study

19 meshes with different element sizes for 3 randomly selected participants were created to identify if the mesh size influences the results of the study.

Identical analysis (base geometry from STL, loading condition, material properties) were performed with meshes with element sizes of 0.5 mm to 5 mm increased by 0.25 mm steps.

Analysis with an element size of 0.5 mm were impossible to solve with the available computer (Intel® Core™ i7-8700 Processor (6 cores), 32 GB RAM) due to too large finite element (FE) models. These meshes included approximately 2 million nodes and 1.9 million elements.

Analysis with too big elements did not converge ("d.n.c."). Big elements resulted in holes within the cortical shaft (Figure 1) leading to "too weak" models for the loading conditions. Since bone marrow has a Youngs' modulus of 1, the cortical bone of the shaft is fully responsible to resist the loading. The holes inside this part makes this impossible leading to negative jacobian elements under loading. This was observed for all meshes with element sizes above 2.5 mm, 3.5 mm and 3.75 mm for in participant 1, participant 2 and participant 3, respectively.

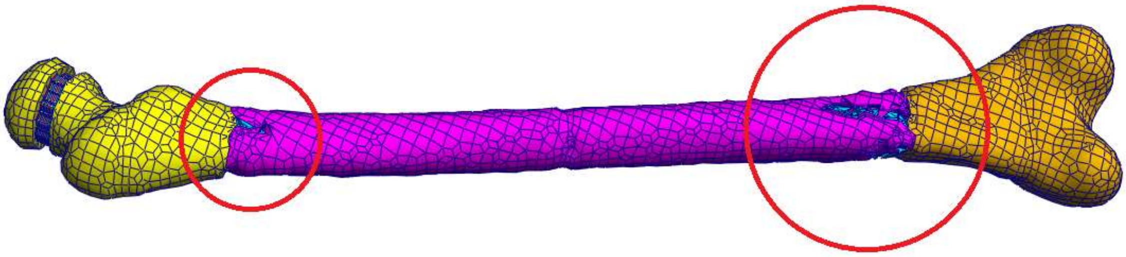

Figure 1. Example of a coarse mesh with holes within the cortical shaft.

Analyzed results of the FE models were number of elements, computation time, maximum displacement and maximum effective stress within the layer of the growth plate which was analyzed during this study (Table 1, **Figure 2** – **Figure 5**). Furthermore, osteogenic index was calculated and projected on the transverse plane (**Figure 6** – **Figure 8**) and the regions with the highest median values were identified (Table 2). Osteogenic index distribution is represented using a blue to red color scheme representing high and low values, respectively. This was performed identically as for all analysis of the full paper. Please read the full paper for detailed information.

The computation time increased significantly for meshes with element sizes below 1 mm. Only minor changes were observed for maximum effective stresses within the layer of growth and the maximum displacement.

An inconsistency for the occurrence of the region with the highest median values was observed at a mesh size of 2 mm and 2.5 mm for participant 3 and 2, respectively. The region with the highest median value was equivalent for all meshes of participant 1.

By visual comparison of the projected osteogenic index it can be seen, that the general shape and distribution is similar between meshes (**Figure 6** – **Figure 8**). With decreasing mesh size, more details can be noticed. However, for meshes with element sizes of 1.75 mm and below, only very little details change.

These results indicate that a mesh size of 1.5 mm is sufficient to identify stresses within the growth plate and calculate the osteogenic index. A mesh size of 1.5 mm seems to be a good compromise between computational time and results.

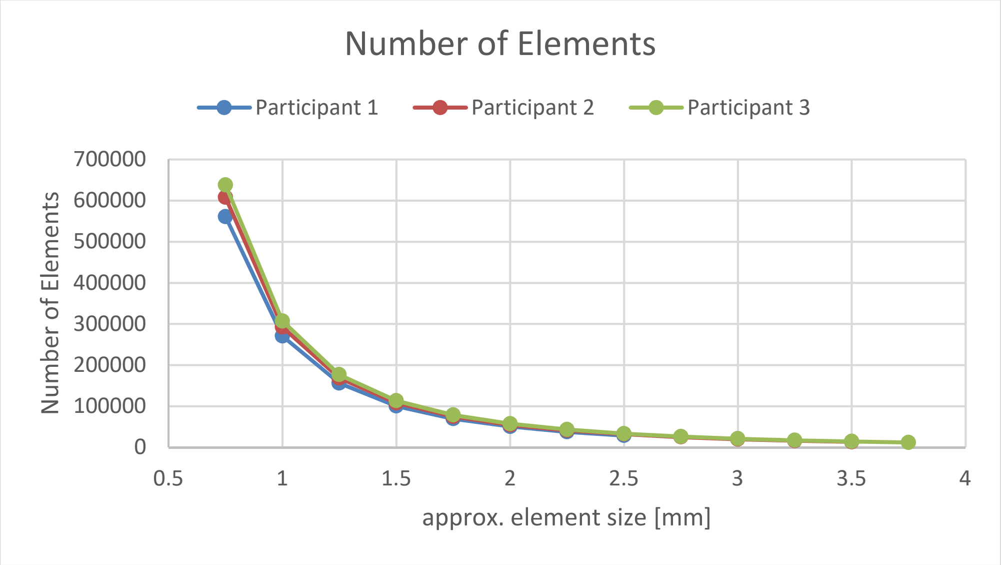

Figure 2. Shows the correlation between mesh size and number of elements of the FE model

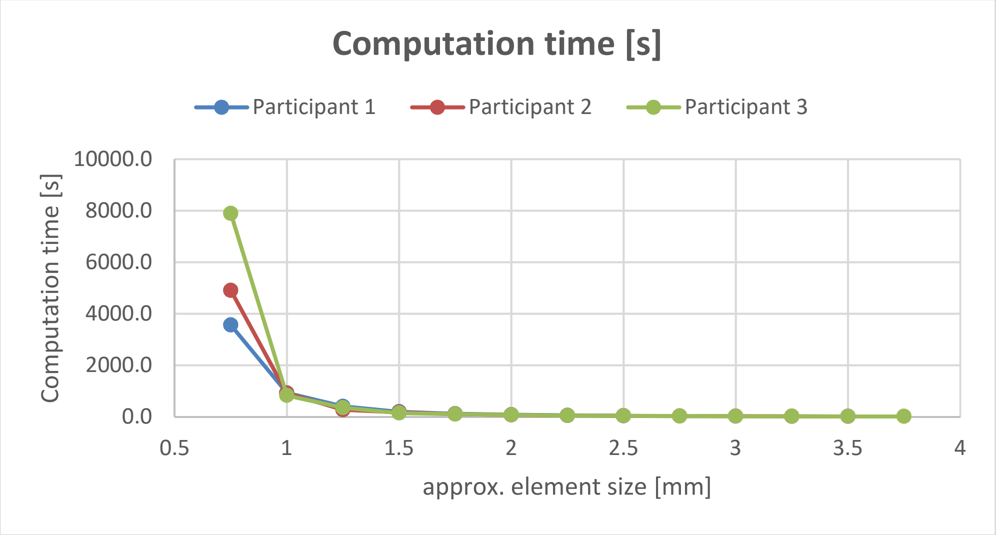

Figure 3. Shows the correlation between mesh size and computation time of the FE model

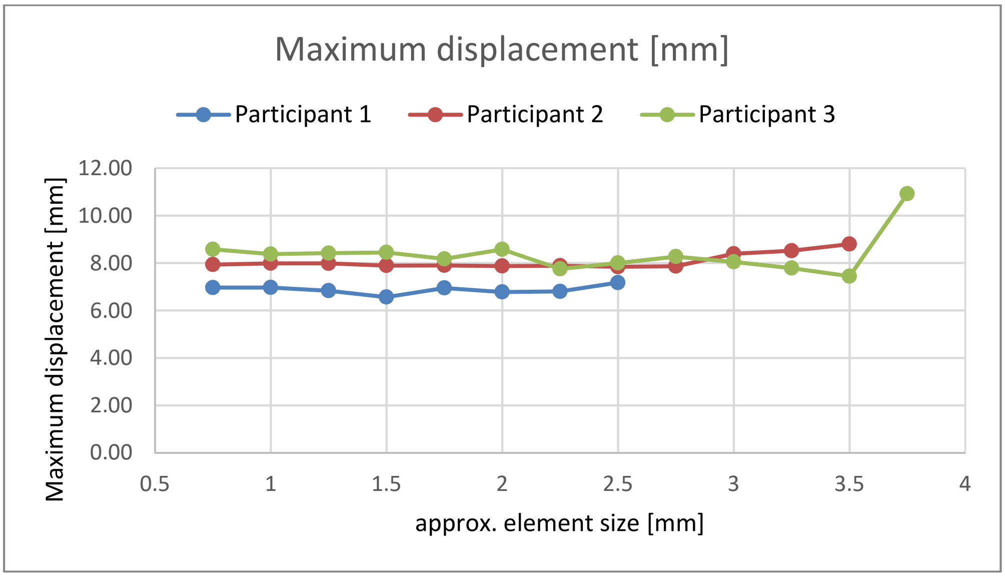

Figure 4. Shows the correlation between mesh size and maximum displacement of the FE model

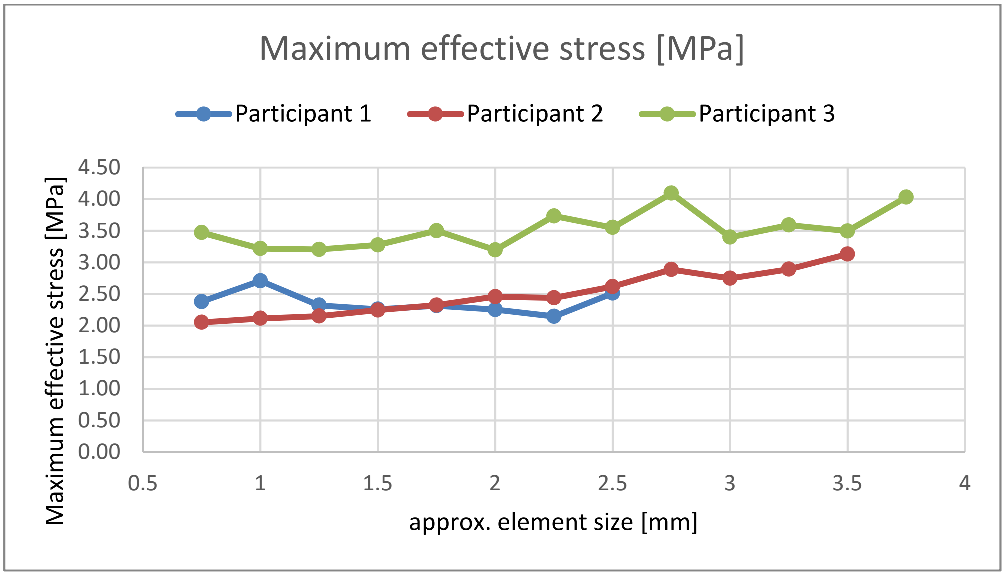

Figure 5. Shows the correlation between mesh size and maximum effective stress within the layer of the growth plate which was analyzed during this study

Table 1:Properties and results of the FE models depending on mesh size

Number of elements													
**Element size**	**0.75**	**1**	**1.25**	**1.5**	**1.75**	**2**	**2.25**	**2.5**	**2.75**	**3**	**3.25**	**3.5**	**3.75**
**Participant 1**	560 795	270 599	156 145	100 425	69 699	50 954	37 948	28 946	d.n.c.	d.n.c.	d.n.c.	d.n.c.	d.n.c.
**Participant 2**	607 984	292 495	168 638	108 138	74 956	54 696	41 408	32 429	25 057	20 007	16 357	13 574	d.n.c.
**Participant 3**	637 958	307 165	176 550	113 467	78 655	57 536	43 437	33 483	26 412	21 047	17 147	14 430	12 040

Maximum displacement [mm]													
**Element size**	**0.75**	**1**	**1.25**	**1.5**	**1.75**	**2**	**2.25**	**2.5**	**2.75**	**3**	**3.25**	**3.5**	**3.75**
**Participant 1**	6.97	6.98	6.84	6.57	6.96	6.79	6.81	7.18	d.n.c.	d.n.c.	d.n.c.	d.n.c.	d.n.c.
**Participant 2**	7.94	7.99	7.99	7.90	7.91	7.88	7.89	7.85	7.87	8.40	8.53	8.80	d.n.c.
**Participant 3**	8.58	8.38	8.42	8.46	8.18	8.58	7.76	8.01	8.28	8.05	7.79	7.45	10.92

Maximum effective stress [MPa]													
**Element size**	**0.75**	**1**	**1.25**	**1.5**	**1.75**	**2**	**2.25**	**2.5**	**2.75**	**3**	**3.25**	**3.5**	**3.75**
**Participant 1**	2.38	2.71	2.32	2.26	2.31	2.25	2.15	2.51	d.n.c.	d.n.c.	d.n.c.	d.n.c.	d.n.c.
**Participant 2**	2.05	2.11	2.15	2.25	2.32	2.46	2.44	2.62	2.89	2.75	2.89	3.13	d.n.c.
**Participant 3**	3.47	3.22	3.20	3.27	3.50	3.20	3.73	3.55	4.09	3.40	3.59	3.50	4.03

Computation time [s]													
**Element size**	**0.75**	**1**	**1.25**	**1.5**	**1.75**	**2**	**2.25**	**2.5**	**2.75**	**3**	**3.25**	**3.5**	**3.75**
**Participant 1**	3562.2	925.7	405.4	197.4	118.1	82.6	57.7	43.7	d.n.c.	d.n.c.	d.n.c.	d.n.c.	d.n.c.
**Participant 2**	4907.1	912.7	275.5	176.5	108.9	71.5	51.6	38.6	29.5	25.8	19.7	17.6	d.n.c.
**Participant 3**	7892.3	831.6	355.4	147.7	108.7	73.0	52.4	41.3	31.8	24.5	24.8	18.6	17.3

Table 2. Occurrence of regions with the highest median values. Orange cells indicate differences to finest mesh (0.75mm)

Region with highest median osteogenic index													
**Element size**	**0.75**	**1**	**1.25**	**1.5**	**1.75**	**2**	**2.25**	**2.5**	**2.75**	**3**	**3.25**	**3.5**	**3.75**
**Participant 1**	Posterior	Posterior	Posterior	Posterior	Posterior	Posterior	Posterior	Posterior	d.n.c.	d.n.c.	d.n.c.	d.n.c.	d.n.c.
**Participant 2**	Medial	Medial	Medial	Medial	Medial	Medial	Posterior	Medial	Posterior	Medial	Medial	Medial	d.n.c.
**Participant 3**	Medial	Medial	Medial	Medial	Medial	Lateral	Medial	Medial	Medial	Lateral	Medial	Medial	Medial

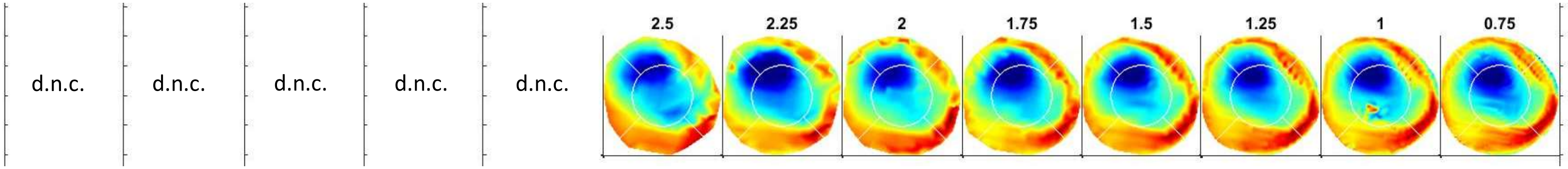

Figure 6. Projected osteogenic index on transverse plane with different mesh sizes (decreasing from left to right) of participant 1

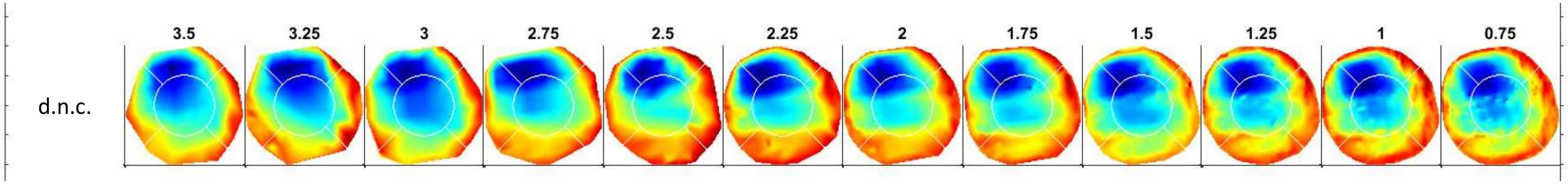

Figure 7. Projected osteogenic index on transverse plane with different mesh sizes (decreasing from left to right) of participant 2

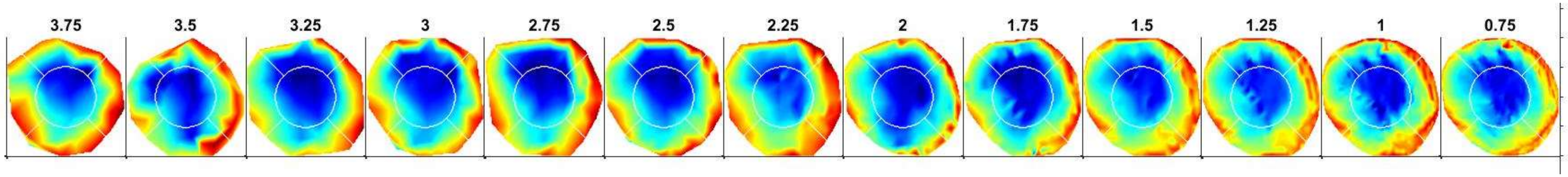

Figure 8. Projected osteogenic index on transverse plane with different mesh sizes (decreasing from left to right) of participant 3
